# Acknowledgement to Reviewers of *Antioxidants* in 2018

**DOI:** 10.3390/antiox8010017

**Published:** 2019-01-09

**Authors:** Antioxidants Editorial Office

**Affiliations:** MDPI, St. Alban-Anlage 66, 4052 Basel, Switzerland

Rigorous peer-review is the corner-stone of high-quality academic publishing. The editorial team greatly appreciates the reviewers who contributed their knowledge and expertise to the journal’s editorial process over the past 12 months. In 2018, a total of 189 papers were published in the journal, with a median time to first decision of 15 days and a median time to publication of 40 days. The editors would like to express their sincere gratitude to the following reviewers for their cooperation and dedication in 2018:
Abrahamsson, ThomasLee, Jin-KooAdam, VojtěchLee, Meng-shiouAfolayan, Adeleye J.Lee, Tzong-ShyuanÅkerström, BoLee, Yi-JangAlmajano, María PilarLehmann, ChristianAltemimi, AmmarLeicht, ChristofAmaya, EnriqueLenaz, GiorgioAmoaku, Winfried M.Lescure, AlainAmorós, AsunciónLevine, RodneyAngelino, DonatoLi, YonghuiAnsorena, DianaLichtenberg, DovAntognelli, CinziaLiddell, Jeffrey R.Arguelles, SandroLin, Pei-HuiAriga, KatsuhikoLingner, JoachimArmand, MartineLiu, JunAudi, SaidLoboda, AgnieszkaAvato, PinarosaLogan, MargaretAvila-Román, JavierLoizzo, Monica RosaAye, YimonLorenzo Rodriguez, Jose ManuelAykin-burns, NukhetLu, JunBachschmid, Markus MichaelLyons, GrahamBaker, JulienMaggini, SilviaBaldisserotto, AnnaMaier, PatrickBallmann, ChristopherMailloux, RyanBanerjee, KalpitaMali, VishalBarba, Francisco J.Malli, RolandBarbagallo, DavideMandrich, LuigiBarbaric, MonikaManjunath, ManuboluBarbe, Mary F.Marchuk, Douglas A.Bárcena, José AntonioMarcinek, David J.Bartlett, David B.Marengo, BarbaraBattaglino, RicardoMarsillach, JuditBauer, GeorgMartin, KeithBeharry, Kay D.Martínez, SidoniaBellés, José MaríaMasek, AnnaBellner, LarsMatsufuji, HiroshiBendich, AdrianneMaupin-Furlow, JulieBeresford, TomMaxwell, Jessie R.Bernini, RobertaMcBean, Gethin J.Blumberg, JeffreyMcDaneld, Tara G.Boggia, RaffaellaMcKenzie, MikeBöhm, VolkerMcPherson, Nicole O.Bonomini, FrancescaMears, JasonBorer, Katarina T.Mendonca, AntonioBoschi-Muller, SandrineMeng, XiaoliBrash, AlanMessina, SamanthaBrewer, Alison C.Meyer, AndreasBrodzki, PiotrMézes, MiklósBubb, Kristen J.Michels, Alexander J.Buchanan, BobMiguel, MariaBudryn, GrażynaMiller, YuryCairrão, ElisaMinetti, GiampaoloCalani, LucaMiranda, Jose M.Campbell, Fiona M.Mita, GiovanniCanabady-Rochelle, Laetitia L.S.Mitra, KatsuriCanini, AntonellaMolina-Montes, EstherCaradonna, FabioMonore, JonCardoso, SusanaMontenegro, LuciaCarey, AmandaMookerjee, ShonaCarnevale, RobertoMoreno, Juan J.Carocho, MarcioMoro, LoredanaCarr, AnitraMorton, DavidCarvalho, DanielMotohashi, KenCastro-Caldas, MargaridaMulas, MaurizioCavallaro, GiacomoMüller-Schüssele, StefanieCejudo, Francisco J.Mullins, David W.Chaiswing, LuksanaMuzykantov, VladimirChakraborty, MallinathNaftalin, RichardChang, Hsueh-WeiNagy, PeterChao, Wen-WanNair, VimalChatterjee, JhinukNakamaru-Ogiso, EikoChen, LinNawirska-Olszańska, AgnieszkaChen, MinNeilson, AndrewChen, Yung-HsiangNguyen, Duc ThanhCherkaoui-Malki, MustaphaNikkanen, LauriCherniack, Evan PaulNomikos, TzortzisChiang, Hsiu-MeiOhnishi, MasatoshiChilibeck, PhilOhta, ShinjiChiu, JoyceOlejar, Kenneth J.Chobot, VladimirOliveira, RuiChohan, MagaliOniga, IlioaraChristides, TatianaOrian, LauraCiaccio, MarcelloOudemans-van Straaten, HeleenÇiçek, Serhat SezaiPace, PaulCiofani, GianniPaixao Coelho, Jose AugustoCiofi-Baffoni, SimonePająk, PaulinaCione, ErikaPaller, ChanningČipak Gašparović, AnaPalma, MiguelColeman, Mitchell C.Panagiotidis, MihalisContino, MarialessandraPappas, ChristosCorino, CarloPark, SunghunCosta, Lucio G.Parsonage, DerekCraige, Siobhan M.Patrice, ThierryCuffe, JamesPedan, VasilisaCumming, Kristoffer T.Peng, Ying-JieDa Silva, Robin P.Pennock, NathanDachs, GabiPereira-da-Silva, LuisDalessandro, GiuseppePerez Gregorio, Maria RosaDall'Acqua, StefanoPeskin, AlexanderDas, Kumuda C.Petrou, Athinoula L.Dasgupta, SantanuPetruzzella, VittoriaDavid, LuminiţaPick, EdgarDavies, MichaelPignitter, MarcDavinelli, SergioPiluso, SusannaDe Filippis, VincenzoPina Pérez, Maria ConsueloDe La Vega, LaureanoPinto, Diana C. G. A.De Las Heras, JavierPiomboni, PaolaDe Mendonça, Dina I. M. D.Plastina, PierluigiDe Vendittis, EmmanuelePlotnikov, EgorDeli, Chariklia K.Pochet, RolandDeSmet, StefanPoplawski, TomaszDiakowska, DorotaPoulsen, Henrik EnghusenDietz, Karl-JosefPrentice, HowardDoles, Jason D.Proestos, CharalamposDonato, RosarioQuinzii, Catarina MariaDonno, DarioRachek, LyudmilaDoriano Bianco, ArmandoRajauria, GauravDubrova, YuriRamachandran, AnupDudas, JozsefRamaraj, PanduranganDüfer, MartinaRambold, Angelika S.Edge, RuthRampon, ChristineEkiert, HalinaRanzato, EliaElhabiri, MouradRapeanu, GabrielaEmmert, SteffenRayner, BenEspinosa-Diez, CristinaReddy, HemachandraEsposito, CiroReukov, VladimirFan, YichongRevilla, EugenioFang, Ronnie H.Rex, ToniaFeresin, Rafaela G.Ribeiro, Maria HFernandes, Ana S.Ricci, AriannaFielding, AlistairRobberecht, HarryFinno, Carrie J.Rodriguez-Cueto, CarmenFiorino, FerdinandoRodriguez-Mateos, AnaFlack, Kyle D.Romani, AndreaFlorencio, Francisco J.Rose, PeterFrank, SašaRuiz-Aceituno, LauraFranken, Nicolaas A. P.Ruperti, BenedettoFriedman, HayaSaada, AnnFrolov, AndrejSafe, StephenFuchs, DietmarSahrawy, MariamFurdui, Cristina MariaSahu, Ravi PrakashGabai, GianfrancoSaisho, YoshifumiGalea, IanSakurai, ToshihiroGalic, AnteSandri, Brian J.Galvano, FabioSanjust, EnricoGan, RenyouSantamaria, RitaGanini, DouglasSantulli, GaetanoGarcia-Castineiras, SixtoSaraiva, NunoGarcía-Moreno, ValmeSastre, JuanGarten, RyanSaturnino, CarmelaGasparrini, MassimilianoSavion, NaphtaliGaucher, CarolineSawicki, RafalGeest, Bart DeSchäfer-Korting, MonikaGentile, FabrizioSchiraldi, ChiaraGeorgakilas, AlexandrosSchlaepfer, Isabel RGeorgopoulos, NikSchmidt, Edward E.Geraghty, PatrickSchöttker, BenGerber, Leonard E.Schröder, KatalinGibson, Gary E.Schulze, JohannesGillis, KeithScicchitano, PietroGiovinazzo, GiovannaSenbonmatsu, TakaakiGizdavic-Nikolaidis, MarijaSeo, DaisukeGolestaneh, NadySerrano, MariaGolmohamadi, AmirSerrano, NúriaGong, XiaomingSetzer, William N.Gonzalez, Michael J.Shao, BaohaiGonzález, SoniaShaw, PeterGonzález-Gallego, JavierSheehy, Paul A.Gorgan, LucianShi, Vivian Y.Goua, MarieShiao, Young-JiGreenberger, Joel S.Sholkamy, EmanGreenlief, MichaelShu, XinhuaGrimm, BernhardSieber, FritzGrimm, Marcus O. W.Signorelli, Salvatore SantoGruhlke, MartinSilswal, NeerupmaGrunig, GabrieleSinanoglou, VassiliaGuido, Luis F.Singh, Dhirendra PratapGullón, BeatrizSingh, VineetHaarmann-Stemmann, ThomasSiristatidis, CharalamposHägglund, Per MårtenSkovsted, Gry FrejaHampel, DanielaSlominski, AndrzejHampton, MarkSmith, Gaynor AnnHan, JaehongSoica, CodrutaHandzlik, JadwigaSoveral, GraçaHardingham, Jennifer E.Stagos, DimitriosHargreaves, Iain P.Stefanidou, Maria E.Harper, JamesStobdan, TseringHarrison, FionaStupka, NicoleHart, JoanneStushnoff, CecilHatano, TsutomuSubramanian, VeedamaliHempel, NadineSuomela, Jukka-PekkaHernández-Hernández, ÁngelSuzuki, TakafumiHisabori, ToruSuzuki, Yuichiro J.Hitosugi, TaroSyvänen, StinaHo, Shin-LonSzumny, AntoniHodge, AllisonTaira, JunseiHossain, MohammadTarrago, LionelHu, GuoliTaticchi, AgneseHuang, HaoTavazzi, BarbaraIametti, StefaniaTaylor, CarolineIbrahim, Salam A.Teiber, JohnIentile, RiccardoTeng, BoIkegami, TakahisaTeschke, RolfIrakli, MariaThierbach, RenéIskandar, Michele M.Thiyam-Holländer, UshaIwaoka, MichioThornton, M. JulieJadavji, Nafisa M.Tiidus, PeterJanda, ElzbietaTipple, Trent E.Jaradat, NidalTokatlidis, KostasJara-Palacios, María JoséTomasetti, MarcoJay-Gerin, Jean-PaulTooyama, IkuoJia, KailiangTraustadόttir, TinnaJohnston, CarolTravers, Jeffrey BryantJones, HuwTsuji, PetraJordão, António ManuelTufarelli, VincenzoKamariah, NeelagandanUgalde, José ManuelKang, Jae SeungUrfer, SilvanKang, Sang WonVaiman, DanielKao, Shu-HueiValentová, KateřinaKappus, RebeccaValgimigli, LucaKaras, MonikaVallesi, AdrianaKarner, CourtneyVallini, GiovanniKarplus, AndrewVan Der Laarse, Willem J.Kashfi, KhosrowVan Hecke, ThomasKato, Takamitsu A.Vashisth, TriptiKauppinen, AnuVaz, JosianaKawakita, HidetakaVázquez-Medina, José PabloKawasumi, MasaokiVeal, Elizabeth A. Kaya, AlaattinVeeramani, SureshKe, Liang-YinVeskoukis, Aristidis S.Kelley, EricVesoulis, Zachary A.Khan, MahmoodVitiello, PeterKhatib, SolimanVon Knethen, AndreasKim, Hwa-YoungWada, JunKim, Hyung SikWang, Horng-darKim, In-JungWang, XinkunKim, Ji YeonWang, XinyuKim, KiyoungWeber, JohnKipp, Anna PatriciaWei, GangKishimoto, YoshimiWeng, Ching-FengKlar, AgnesWest, James D.Klarskov, KlausWigle, JeffreyKlimis-Zacas, DorothyWilkinson, BrianKocot, JoannaWilliams, Christopher C.Kontogiorgis, ChristosWilliams, Christopher S.Koopman, ReneWilson, JohnKoshiba, TakumiWilson, Mark AKotula-Balak, MalgorzataWojtunik-Kulesza, Karolina A.Koulen, PeterWood, ScottKowalczewski, PrzemysławWoodman, TimKraemer, Brian C.Wu, Li-ChenKrajka-Kuźniak, ViolettaWu, Ming-JiuanKrupa-Kozak, UrszulaXiaoli, AlusKuhn, HartmutXin, LingKukula-Koch, WirginiaYi, Tae HooKumar, BinodZachut, MayakKump, DavidZanoli, LucaKundur, Avinash ReddyZarrelli, ArmandoKurdowska, Anna K.Zeman, Daniel MeierLance, BaumgardZhang, HuaLatella, GiovanniZhang, PangzhenLaVoy, EmilyZhou, Huanjiao JennyLe, Thu H.Zlotek, UrszulaLebaron, RichardZou, PingLedgerwood, Elizabeth C.Zuber, PeterLee, Byeong JaeZunin, PaolaLee, Jae WookŻyżelewicz, Dorota

